#  Morphological and functional carotid vessel wall properties in relation to cerebral white matter lesions in myocardial infarction patients

**DOI:** 10.1007/s12471-015-0693-6

**Published:** 2015-05-12

**Authors:** E.S.J. Kröner, J. van der Grond, J.J.M. Westenberg, E.E. van der Wall, H.-M.J. Siebelink, H.J. Lamb

**Affiliations:** 1 Department of Cardiology, Leiden University Medical Center, Albinusdreef 2, 2333 ZA Leiden, The Netherlands; 2 The Interuniversity Cardiology Institute of the Netherlands, Utrecht, The Netherlands; 3 Department of Radiology, Leiden University Medical Center, Leiden, The Netherlands

## Abstract

**Objective:**

Atherosclerotic *large* vessel disease is potentially involved in the pathogenesis of cerebral *small* vessel disease related to occurrence of white matter lesions (WMLs) in the brain. We aimed to assess morphological and functional carotid vessel wall properties in relation to WML using magnetic resonance imaging (MRI) in myocardial infarction (MI) patients.

**Materials and methods:**

A total of 20 MI patients (90 % male, 61 ± 11 years) underwent carotid artery and brain MRI. Carotid vessel wall thickness (VWT) was assessed, by detecting lumen and outer wall contours. Carotid pulse wave velocity (PWV), a measure of elasticity, was determined using the transit-time method. Patients were divided according to the median VWT into two groups. Brain MRI allowed for the WML score.

**Results:**

Mean VWT was 1.41 ± 0.29 mm and mean carotid PWV was 7.0 ± 2.2 m/s. A significant correlation (Pearson r = 0.45, *p* = 0.046) between VWT and PWV was observed. Furthermore, in the group of high VWT, the median WML score was higher as compared with the group with lower VWT (4.0 vs 3.0, *p* = 0.035).

**Conclusions:**

Carotid artery morphological and functional alterations are correlated in MI patients. Patients with high VWT showed a higher amount of periventricular WMLs. These findings support the hypothesis that atherosclerotic *large* vessel disease is potentially involved in the pathogenesis of cerebral *small* vessel disease.

## Introduction

Vascular risk exposure and age exert systemic effects on both the morphology and function of the arterial vessel wall by degeneration of the aortic media and breakdown of elastic fibres [1]. Accordingly, in patients with established atherosclerotic disease, e.g. patients with a previous myocardial infarction (MI), accelerated morphological changes are considered to be associated with increased arterial stiffness. Moreover, atherosclerotic *large* vessel disease is potentially involved in the pathogenesis of cerebral white matter lesions (WMLs) that are generally regarded as manifestations of cerebral *small* vessel disease [2–4]. Indeed, it is hypothesised that pathologically increased arterial stiffness results in deficient absorption of the pulse wave travelling through the vascular system. A deficient absorption of the systolic pulse wave may result in transmission of high pulsatile flow from the aorta towards the carotid artery and the brain, potentially initiating carotid vessel wall remodelling and functional changes which may lead to the development of cerebral small vessel disease and subsequent WMLs. Accordingly, the association between systemic *large* vessel disease, cerebral *small* vessel disease and WMLs in MI patients is of interest.

Noninvasive evaluation of morphological and functional vessel wall properties and cerebral WMLs is feasible using a comprehensive magnetic resonance imaging (MRI) approach [5–7]. Still, very little is known about the direct association between carotid vessel wall thickness (VWT), vascular stiffness and white matter brain lesions in MI patients. Therefore, the purpose of this study was to assess the association between morphological and functional carotid vessel wall properties and cerebral periventricular WML in MI patients using MRI.

## Materials and methods

### Population and study protocol

**Fig. 1:**
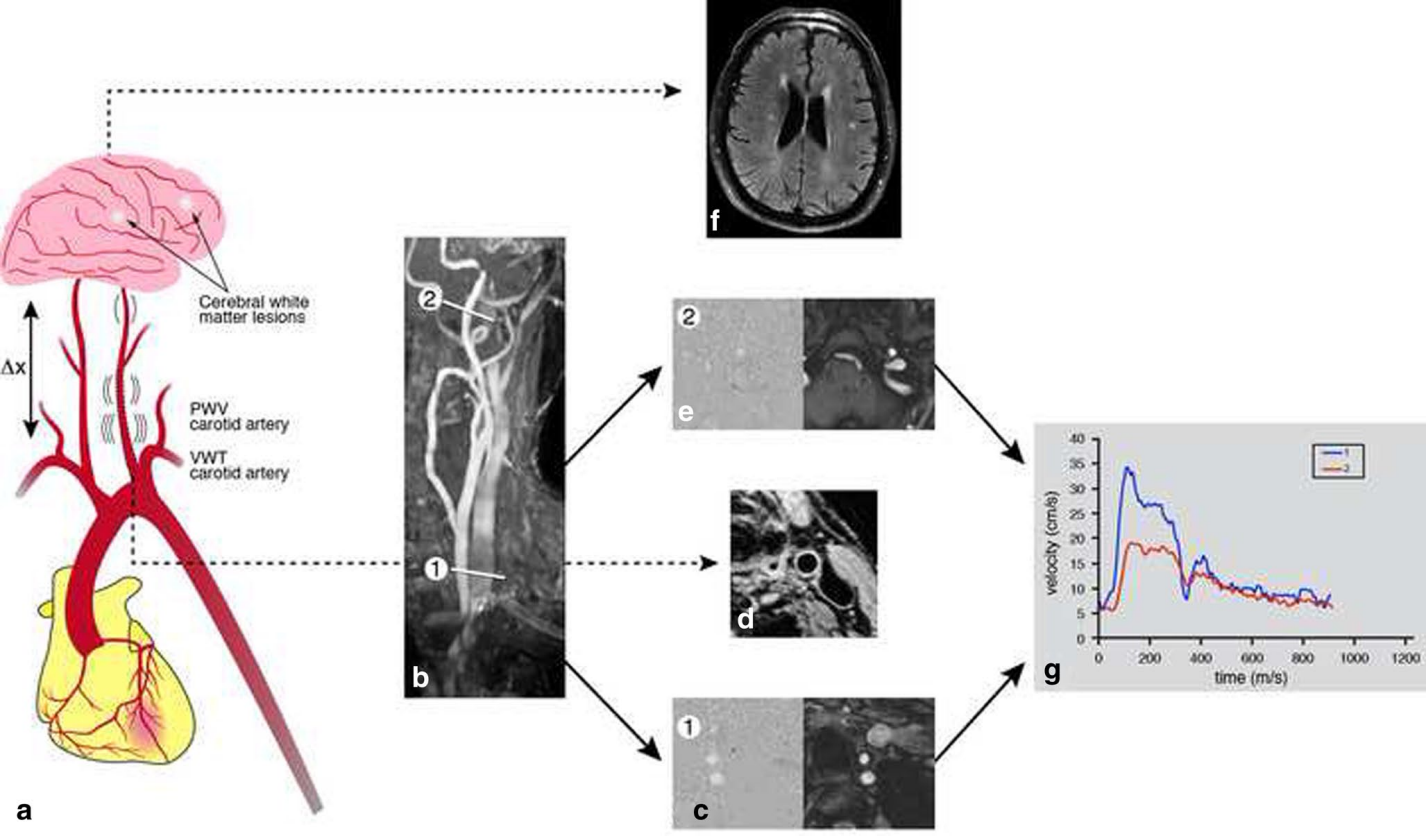
A schematic representation of the study protocol (**a**). Carotid vessel wall properties (vessel wall thickness (VWT) and pulse wave velocity (PWV)) and cerebral periventricular white matter lesions (WML) were assessed in MI patients. PWV was assessed at two locations, proximally at the left common carotid artery just above the aortic arch (1) and distally just below the petrous portion of the left internal carotid artery (2), which were planned on the rotational maximum-intensity projection of a 3D time-of-flight acquisition of the carotid arteries (**b**). The corresponding velocity-encoded images are represented in **c, e** for the proximal and distal acquisition respectively. From the propagation of the velocity waveforms (**g**), PWV is determined. VWT was assessed at the left common carotid artery (4-mm proximal to the carotid bifurcation) (**d**). Cerebral WML were determined using spin-echo T2-weighted and a fluid-attenuated inversion recovery (**f**).

A total of 20 MI patients (90 % male, 61 ± 11 years) who previously suffered from an MI were included. Approval from the local medical ethics committee was obtained and all patients gave written informed consent. Patients underwent 3 T MRI examinations (Philips Achieva Philips Medical Systems, Best, the Netherlands) between October 2011 and November 2012. Carotid artery VWT, pulse wave velocity (PWV) in the carotid artery and cerebral WMLs were assessed using MRI techniques [7–9] (Fig. 1).

### MRI acquisition

#### Carotid vessel wall thickness

Carotid VWT was determined using a two-dimensional (2D) T1-weighted segmented gradient echo sequence using a standard Philips SENSE-flex-M surface coil with two flexible elements of 14 × 17 cm as previously described [5]. Scan parameters: 2D black blood (BB) T1-weighted fast gradient echo sequence, field of view (FOV) 140 × 140 mm^2^, 2.0 mm slice thickness, flip angle (FA) 45º, repetition time (TR) 12.4 ms, echo time (TE) 3.5 ms, acquired resolution 0.46 × 0.46 × 2.0 mm^3^, number of signal averages (NSA) 2. Acquisitions were gated at end-diastole using vector ECG.

#### Carotid pulse wave velocity

Carotid PWV was assessed by two consecutive velocity-encoded (VE) MRI acquisitions using a 16-element neurovascular head-neck coil, as previously described [8]. The first acquisition (proximal) was planned perpendicular to the left carotid artery at the level of the origin of the common carotid artery and the second (distal), at the level of the internal carotid artery, below the petrous segment (Fig. 1b, c, e).

Scan parameters were: FOV 200 × 200 mm^2^, slice thickness 5 mm, FA 10°, TR 6.2 ms, TE 3.4 ms, acquisition resolution 1.52 × 1.50 × 5.0 mm^3^, NSA 1, velocity sensitivity (V_enc_) for the proximal acquisition 150 cm/s and distal acquisition 120 cm/s, both in through-plane direction. The true temporal resolution (TRes, defined as 2 × TR, amounted to 12.4 ms per heart phase). Retrospective gating using vector ECG triggering was used.

#### Cerebral white matter lesions

Cerebral WMLs were determined using spin-echo T2-weighted and fluid-attenuated inversion recovery (FLAIR) sequences as previously described [10]. Scan parameters for T2-weighted imaging: FOV 224 × 180 mm^2^, matrix size 448 × 320, 40 transverse slices without gap, 3.6 mm slice thickness, FA 90°, TR 4200 ms, TE 80 ms. Scan parameters for FLAIR sequence: FOV 220 × 176 mm^2^, matrix size: 320 × 240, 25 transverse slices without gap, 5 mm slice thickness, FA 90°, TR 11000 ms, TE 125 ms [10].

### Image analysis

#### Carotid vessel wall thickness

Contour segmentation was performed on a slice of the left common carotid artery (4-mm proximal to the carotid bifurcation) using Vessel MASS software (Leiden University Medical Centre, Leiden, the Netherlands) as previously described [11] (Fig. 1d). Mean and maximal carotid vessel wall thickness (mm) were evaluated. Contour segmentation was performed by a researcher with 3 years of experience in cardiac MRI.

#### Pulse wave velocity

Carotid PWV was obtained from the VE MRI data [7, 8]. The carotid artery path length (∆x) between subsequent proximal and distal carotid artery sampling sites was manually determined. Wave propagation was evaluated from maximal velocity time curves that were obtained by using FLOW software (Leiden University Medical Centre, Leiden, the Netherlands) with automated contour detection for image segmentation. The foot-to-foot definition was used for ∆t (i.e. the transit time) assessment; with automated detection of the foot of the systolic velocity wave front (i.e. the wave arrival time). Accordingly, carotid PWV was calculated as ∆x/∆t (m/s) (Fig. 1g).

#### White matter lesions

White matter hyperintensities/lesions were defined as areas within the cerebral white matter, with increased signal on both T2-weighted images and FLAIR images without mass effect [10] (Fig. 1f). WMLs were rated according to a slightly modified version of the semi-quantitative rating scale of Scheltens et al. [12], as previously described [10]. Periventricular and subcortical WMLs were rated separately. Periventricular WMLs for three separate regions (anterior, lateral, posterior) were scored as: no white matter lesions (0); normal amount of white matter lesions (1); abnormal amount (2); very abnormal amount (3) [12]. Next, a total periventricular WML score was calculated as the sum of the three individual scores.

Subcortical WMLs were scored as: no white matter lesions (0); 1–3 small lesions (1); > 3 small lesions (2); very abnormal, confluent lesions (3) [12].

WML were scored by a researcher (JvdG) with 15 years of experience in neuroradiology.

### Statistical analysis

Statistical analysis was performed using SPSS for Windows (version 18.0; SPSS, Chicago, Illinois, USA). Data are expressed as mean ± standard deviations (SD) unless stated otherwise. The relation between and morphological measurements (VWT in the carotid artery) and functional properties (i.e. PWV of the carotid artery) was assessed. Next, the association between vessel wall parameters and WMLs was investigated. The association between mean and maximal carotid VWT and carotid PWV was assessed using linear regression. Multivariate linear regression analysis with PWV as an independent variable and VWT and age as dependent variables was performed to assess the influence of age on the association between VWT and PWV. Next, patients were divided into groups, according to the median of VWT. Carotid PWV as well as the total amount of periventricular and subcortical WMLs was compared (using the Mann–Whitney test) between the group with high VWT (>median VWT) versus the group with low VWT (≤ median VWT).

## Results

Patient characteristics and results are presented in Table 1. In all, 20 MI patients (18 male, 2 female, mean age 61 ± 11 years) were included. All patients were prescribed medication for secondary prevention of MI (i.e. anticoagulants and or platelet inhibitors, beta-blockers, angiotensin-converting enzyme inhibitors or angiotensin II inhibitors and statins) according to clinical guidelines [13].

**Table 1:** Characteristics and results of study population (*n* = 20)

Age at magnetic resonance imaging (MRI) (years)	61 ± 11
Age range (min.–max.)	(37–82)
Days between MI and vessel wall MRI scan (days)	299 ± 144
Culprit vessel at MI	
Left anterior descending artery	5 (25 %)
Right coronary artery	13 (65 %)
Circumflex artery	2 (10 %)
Peak troponin T values (µg/l)	3.8 ± 2.4
Male gender, n (%)	18 (90 %)
BMI (kg/m^2^)	26 ± 3
Brachial blood pressure (mmHg)	
Systolic	125 ± 23
Diastolic	76 ± 12
Heart rate (beats per minute)	74 ± 16
Patients with arterial hypertension, n (%)	10 (50 %)
Patients with diabetes mellitus, n (%)	2 (10 %)
Smokers, n (%)	8 (40 %)
Total cholesterol (mmol/l)	5.09 ± 1.17

Data are represented as mean ± standard deviation

*MI* myocardial infarction, *BMI* body mass index

### Vessel wall morphology and function

MRI measurements are presented in Table 2. Mean carotid vessel wall thickness was 1.41 ± 0.29 mm and mean carotid PWV 7.0 ± 2.2 m/s.

**Table 2:** Magnetic resonance imaging carotid vessel wall (*n* = 20)

	Myocardial infarction patients (*n* = 20)
Trajectory carotid artery, mm	147 ± 20
PWV carotid artery, m/s	7.0 ± 2.2
Mean vessel wall thickness carotid artery, mm	1.41 ± 0.3
Maximal vessel wall thickness carotid artery, mm	1.73 ± 0.4

Data are represented as mean ± standard deviation

*PWV* pulse wave velocity

**Fig. 2:**
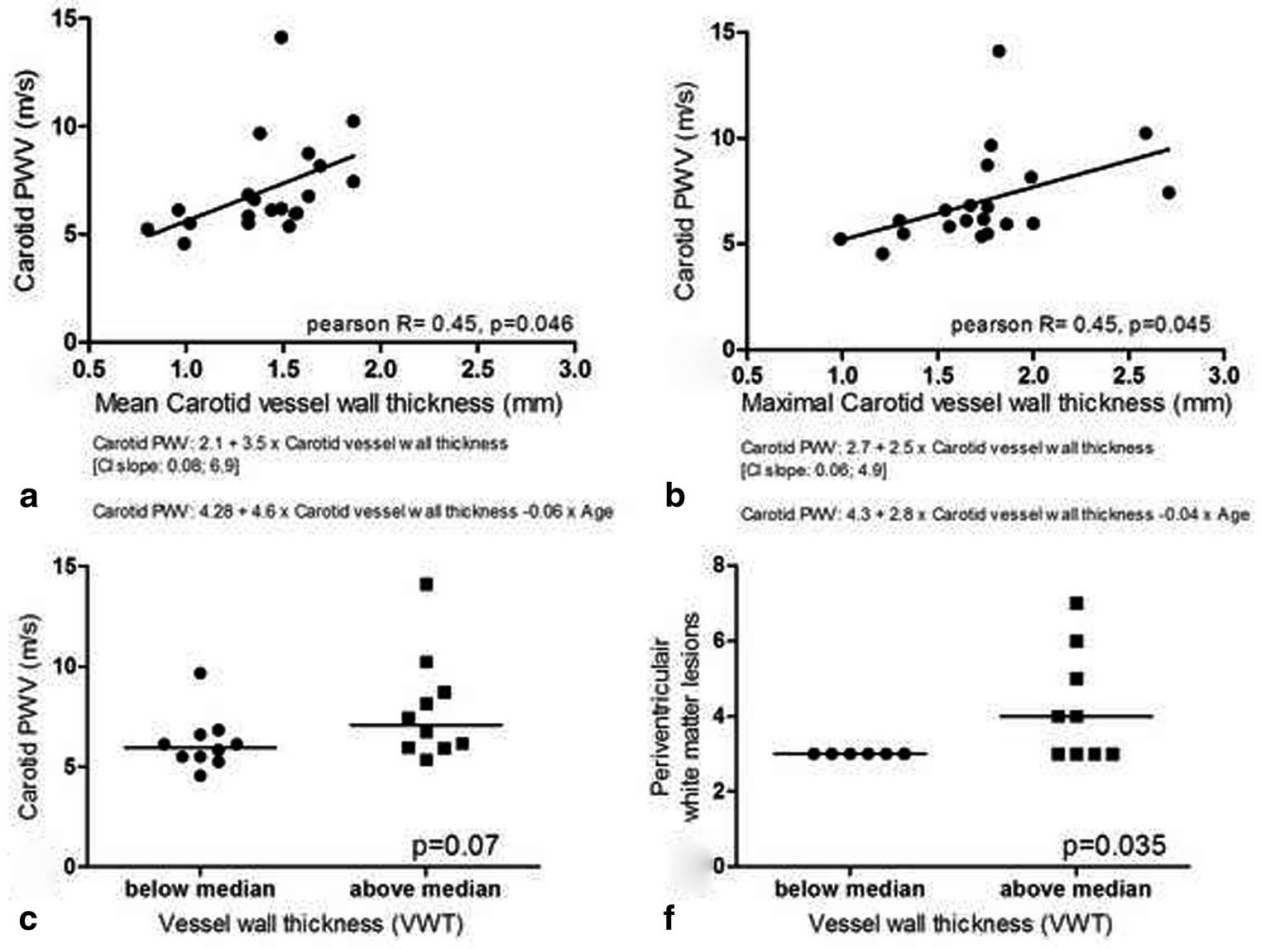
Association between carotid artery pulse wave velocity and mean and maximal carotid vessel wall thickness in myocardial infarction patients (**a, b**). Comparison of carotid pulse wave velocity and white matter lesions in patients with low versus high carotid vessel wall thickness (**c, d**)

Interestingly, PWV of the carotid artery was significantly correlated with VWT of the carotid artery (Pearson *r* = 0.45, *p* = 0.046). The associations between PWV of the carotid artery and mean and maximal VWT are presented in Fig. 2a and b. Also, the influence of age on the association between PWV and VWT is shown. Age sharpened the association between vessel wall thickness and pulse wave velocity. However, age was not a statistically significant predictor in the multiple regression model.

### White matter lesions

In 15 out of 20 patients, brain MRI was also acquired; 5 (33 %) patients showed an abnormal amount of periventricular WMLs (*n* = 5), according to the modified Scheltens score; 12 (80 %) patients were diagnosed with the presence of subcortical WMLs. Detailed scores for anatomical subdivision are provided in Table 3.

**Table 3:** MRI white matter lesions(*n* = 15)

	Periventricular lesion score			Subcortical lesion score
	Anterior	Lateral	Posterior	
Score 0	0 (0)	0 (0)	0 (0)	3 (20)
Score 1	11 (73)	11 (73)	14 (93)	5 (33)
Score 2	3 (20)	3 (20)	1 (7)	6 (40)
Score 3	1 (7)	1 (7)	0 (0)	1 (7)

Data are presented as number (percentage)

### Association vessel wall thickness, pulse wave velocity and white matter lesions

In patients with high VWT (>median VWT), a borderline significantly higher carotid PWV (7.1 m/s versus 5.98 m/s, *p* = 0.07) as compared with the patients with low vessel wall thickness was observed (Fig. 2c). Furthermore, the total periventricular WMLs score was significantly higher in the patients with high vessel wall thickness as compared with patients with low vessel wall thickness (4.0 vs 3.0, *p* = 0.035) (Fig. 2d). In patients with high VWT, the total subcortical WMLs score was similar as compared with patients with low VWT (2.0 vs 1.0, *p* = 0.3).

## Discussion

The present study evaluated the association between carotid morphological and functional imaging parameters and cerebral WML in patients after MI. The main findings of our study are: (i) carotid VWT and carotid PWV are statistically significantly correlated in MI patients, and (ii) the total periventricular WMLs score was higher in the MI patients with high carotid vessel wall thickness versus the patients with low carotid VWT.

To the best of our knowledge, our study is the first to report an evaluation of both carotid VWT and PWV and cerebral WMLs in MI patients using a comprehensive MRI evaluation.

The assessment of VWT and PWV by MRI correlates to presence of WMLs, which is of clinical interest. Previous studies used echo (Doppler) for the assessment of VWT and arterial stiffness [14–16]. This imaging technique is restricted by the choice of imaging plane (i.e. only sampling in the common carotid artery is possible). In contrast, MRI allows direct sampling of VWT and PWV, without restrictions regarding the choice of imaging plane, thereby allowing sampling along the carotid arterial trajectory from the common carotid artery into the internal carotid artery. A recent MRI study by Corti et al. [17] showed a decrement in carotid vessel wall area and maximal carotid artery thickness, but not minimal carotid artery thickness in hypercholesterolaemic patients after statin use. We aimed to explore not only the morphological vessel wall changes but also the association between morphological and functional properties of the vessel wall.

### Vessel wall morphology and function

Our study showed a significant association between mean and maximal VWT and PWV in the carotid artery in patients after MI. Our findings are consistent with a previous population-based cohort study by Van Popele et al. [15], who found increased common carotid stiffness assessed by ultrasound, in the highest quartile of intima-media thickness of the common carotid artery. Interestingly, our results indicate that morphology and functional parameters remain clearly associated in patients with established atherosclerotic disease. We also observed that age influenced the association between VWT and PWV. This finding is in line with previous studies describing increased carotid artery stiffening with age [2, 14]. Of note, in the present study, age was not a statistically significant predictor in the multiple regression model. This could potentially be explained by the relatively small study group. Accordingly, future studies remain needed to further assess the influence of age on the association between vessel wall morphology and function in patients with established atherosclerotic disease.

### White matter lesions

The prevalence of WMLs observed in the present study is in line with previous studies [10, 18]. A high prevalence of WMLs in MI patients is indeed to be expected, since risk factors associated with an MI are also risk factors for cerebral small vessel disease [19].

### Increased vessel wall thickness and white matter lesions

Our study revealed that in MI patients with high VWT, the amount of periventricular WMLs was significantly higher as compared with subjects without increased VWT. Our findings are in line with a previous study in a population-based cohort (*n* = 640, age: 59–71 years), describing the association between carotid atherosclerosis assessed by ultrasonography and WMLs [20]. Moreover, Kwee et al. [21] showed that in transient ischaemic attack (TIA)/stroke patients with carotid artery stenosis, carotid plaque burden and WML severity were associated. Our results indicate that carotid VWT and WMLs are not only associated in the elderly general population and TIA/stroke patients but also in patients with a previous MI.

In contrast to the result for periventricular WMLs, no relation between carotid vessel wall thickness and subcortical WMLs was observed. This difference is in line with a previous study [22] and may be explained to be the result of hypoperfusion that can be caused by large vessel disease. Due to differences in blood supply, the periventricular white matter is more vulnerable to a decrease in cerebral blood flow [10, 22].

The current study showed associations between morphological and functional carotid vessel wall properties and cerebral white matter lesions in MI patients. The presence of WMLs in MI patients may have relevant clinical implications, since WMLs have been suggested to increase the risk of stroke and cognitive decline [18]. However, it is not likely that MR imaging of the brain will be a routine investigation in MI patients. But, for identification of MI patients at risk, carotid vessel wall parameters might become beneficial in the future.

### Limitations

Our study has some limitations. First, it involves a *cross-sectional* design in a relatively small group of patients (*n* = 20), of which 15 underwent brain MRI.

Follow-up studies are needed to further elucidate pathophysiological mechanisms between large vessel atherosclerosis and small vessel disease in patients with established atherosclerotic disease.

Furthermore, the comprehensive MRI evaluation of the present study is time-consuming and relatively expensive in comparison with echocardiography. But for serial assessment of both vessel wall parameters and WML, MRI allows a noninvasive, reproducible evaluation without restriction regarding imaging plane.

### Conclusion

Morphological and functional alterations in the carotid artery are significantly correlated in MI patients. Patients with high carotid VWT showed a higher amount of periventricular WMLs. These findings support the hypothesis that atherosclerotic *large* vessel disease is potentially involved in the pathogenesis of cerebral *small* vessel disease.
